# CCR6 and CXCR6 Identify the Th17 Cells With Cytotoxicity in Experimental Autoimmune Encephalomyelitis

**DOI:** 10.3389/fimmu.2022.819224

**Published:** 2022-02-01

**Authors:** Lifei Hou, Koichi Yuki

**Affiliations:** ^1^ Department of Anesthesiology, Critical Care and Pain Medicine, Boston Children’s Hospital, Boston, MA, United States; ^2^ Department of Anaesthesia and Department of Immunology, Harvard Medical School., Boston, MA, United States

**Keywords:** CXCR6, CCR6, Th17, cytotoxicity, EAE, Multiple Sclerosis

## Abstract

Due to the plasticity of IL-17-producing CD4 T cells (Th17 cells), a long-standing challenge in studying Th17-driven autoimmune is the lack of specific surface marker to identify the pathogenic Th17 cells *in vivo*. Recently, we discovered that pathogenic CD4 T cells were CXCR6 positive in experimental autoimmune encephalomyelitis (EAE), a commonly used Th17-driven autoimmune model. Herein, we further revealed that peripheral CXCR6^+^CD4 T cells contain a functionally distinct subpopulation, which is CCR6 positive and enriched for conventional Th17 molecules (IL-23R and RORγt) and cytotoxic signatures. Additionally, spinal cord-infiltrating CD4 T cells were highly cytotoxic by expressing Granzyme(s) along with IFNγ and GM-CSF. Collectively, this study suggested that peripheral CCR6^+^CXCR6^+^CD4 T cells were Th17 cells with cytotoxic property in EAE model, and highlighted the cytotoxic granzymes for EAE pathology.

## Introduction

Multiple sclerosis (MS) is a devastating autoimmune disease with progressive neurological dysfunction due to demyelination of the central nerve system (CNS) (1). EAE is the most commonly used murine MS model and driven by self-reactive CD4 T cells (CD4 cells thereafter) (1). The disease-inducing CD4 cells were initially thought to be Th1 cells, but later were described as Th17 cells (2, 3). Indeed, Th17 cells themselves are not pathogenic (4), but are converted, under the priming of myeloid cell-derived IL-1 and IL-23 (5–7), into pathogenic CD4 cells, which lose IL-17-producing ability and alternatively produce IFNγ and GM-CSF (8–12). Despite their cytokine profiles, little else is known about the pathogenic CD4 cells, mainly due to the lack of specific marker to precisely identify them *in vivo*.

We previously found SerpinB1 (sb1, Serine Protease Inhibitor B1), an endogenous protease inhibitor (13), to be a signature gene of IL-17-producing γδ T cells (14) and Th17 cells (15). Subsequent studies showed that *sb1-ko* mice were resistant to EAE with a paucity of CXCR6^+^CD4 cells (16), which were highly enriched in the spinal cord, secreted inflammatory cytokine IFNγ and GM-CSF, contained cytotoxic Granzyme-C, and proliferated rapidly (16). Depleting CXCR6^+^CD4 cells by anti-CXCR6 antibody dramatically ameliorated established EAE, confirming the CXCR6^+^CD4 cells as the driver of EAE pathology (16). In this study, we further characterized CXCR6^+^CD4 cells.

## Material and Method

### Mice

*Wild type* C57BL/6J mice (*wild type*, *wt*) were originally purchased from the Jackson laboratory and maintained in an animal facility at the Boston Children’s Hospital. Animal protocols were approved by the Institutional Animal Care and Use Committee of Boston Children’s Hospital. EAE model induction, flow cytometry, cell sorting, q-PCR analysis, cell counting, and RNA sequencing were performed as previously described (16). In brief, to induce EAE, *wt* mice in the C57BL/6J background were injected with MOG_35–55_ emulsified with complete Freund’s adjuvant followed by 200 ng pertussis toxin on days 0 and 2.

### RNAseq Analysis

CD4 effector cells (CD44^+^CD4) were sort-purified from pooled draining lymph node (dLN) cells and spinal cords of MOG-immunized *wt* mice by FACSAria system at the disease onset. The cells were stimulated for 4 h with PMA (50 ng/ml) and ionomycin (750 ng/ml) (Sigma-Aldrich), and RNA was purified using QIAGEN RNeasy Plus Mini kits and quantified by optical density at 260/280/230 nm. TruSeq RNA V2 kits were used to construct transcript-specific libraries that were sequenced on Illumina HiSeq2500. The resulting 4.5 Gb/genotype of raw data was trimmed, and 20 million reads were mapped. Genes that had expression levels (FPKM) ≥1.0 were analyzed for differential expression (https://www.ncbi.nlm.nih.gov/geo/query/acc.cgi?acc=GSE192878)

### Reverse Transcription and qPCR Analysis

CCR6^+^CXCR6^-^CD44^+^CD4, CCR6^+^CXCR6^+^CD44^+^CD4, CCR6^-^CXCR6^+^CD44^+^CD4 were sort-purified from pooled lymph node cells of MOG-immunized *wt* mice by FACSAria system at disease onset. RNA was isolated using RNeasy Plus kits (74134, Qiagen) according to the manufacturer**’**s protocol and reverse-transcribed using the iScript**™** cDNA Synthesis kit (Bio-Rad). The qPCR assays were performed on the CFX96**™** Real-Time System (Bio-Rad) with the iTaq**™** Universal SYBR Green Supermix (Bio-Rad) using 30 s denaturation at 95**°**C and 40 cycles of 5 s at 95**°**C and 30 s at 61**°**C using the primers. Relative expression level for each gene was calculated by using the ^ΔΔ^Ct method and normalizing to *Actb.*

### Flow Cytometry

Cells were stained with fluorochrome-conjugated antibodies to surface markers. Fluorochrome-conjugated antibodies were from Biolegend: FITC- or PE-Cy7-anti-mCD3 (145-2C11), Pacific blue- or PE-anti-mCD45 (30-F11), Pacific blue- or PE-Cy7- or APC- anti-mCD4 (GK1.5), PE-anti-mIL1R1 (JAMA-147), FITC-anti-mCD44 (IM7), APC-anti-mCXCR6 (SA051D1), APC- or PE-Cy7-anti-mCCR6 (29-2L17). From R&D system: PE-anti-mIL-23R (753317). Data were acquired on a Canto II cytometer (BD Biosciences) and analyzed using FlowJo software (Tree Star). Cell counting was achieved by using AccuCount beads (Spherotech).

### CyTOF Assay

After red blood cell lysis, both draining lymph node cells and peripheral blood cells were stimulated for 4 h with PMA (50 ng/ml) and ionomycin (750 ng/ml) (Sigma-Aldrich) in the presence of Brefeldin A. Cells were then collected and resuspended in cell staining buffer (Fluidigm; San Francisco, CA). After centrifugation, Fc receptor blocking reagent (clone 93, Biolegend) was used at a 1:100 dilution in for 10 min, followed by incubation with metal conjugated surface antibodies for 30 min. Then the cells were fixed and permeabilized by using fixation/permeabilization reagents (BD Bioscience). The permeabilization buffer was made by diluting the 10× stock solution (51-2091, BD Biosciences) in UltraPure distilled water (Invitrogen). All antibodies were purchased from the CyTOF core facility at Brigham and Women’s Hospital. DNA was labeled with iridium intercalator solution (Fluidigm). Samples were subsequently washed and reconstituted in Milli-Q filtered distilled water in the presence of EQ Four Element Calibration beads (Fluidigm, catalog 201078). Samples were acquired on a Helios CyTOF Mass Cytometer (Fluidigm) at Cellular Profiling Core Facility (School of Public Health, Harvard Medical School, Boston, MA, USA); Data were analyzed by using spanning-tree progression analysis of density-normalized events (SPADE) and vi stochastic neighbor embedding (SNE) on Cytobank software.

### Statistical Analysis

Statistical analyses were performed using Prism 4 (Graphpad Software). Student’s *t*-test was used. P-values <0.05 were considered significant.

## Results

**Figure 1:**
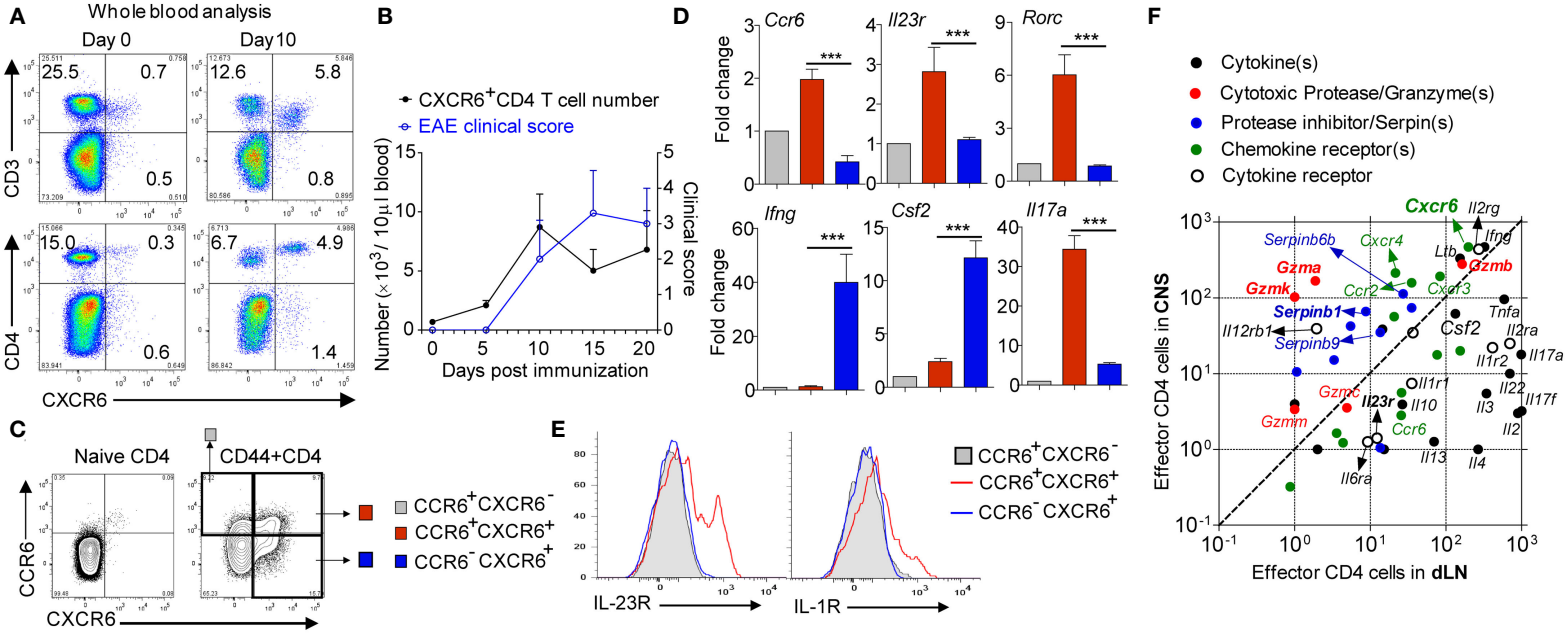
CXCR6+CD4 cells in EAE. **(A)** Representative FACS data gating on blood leukocyte. **(B)** Cell number. N = 4 in each time points. **(C)** Gating strategy for FACS sorting or analysis of CD4 cells in dLN at EAE onset. **(D)** Relative gene expression of CCR6+CXCR6−, CCR6+CXCR6+and CCR6−CXCR6+ effector CD4 cells; CCR6+CXCR6− was used as control. Data are mean ± SEM of 3 independent experiments. ***p <0.001. **(E)** Representative FACS data showing IL23R and IL1R expression on CD4 cells in dLN at EAE onset. **(F)** Effector CD4 cells were isolated from both dLN and spinal cord at EAE onset, and subjected to bulk RNAseq. Data are presented by FMPK.

We immunized the wild type (wt) mice with MOG to induce EAE and monitored the CXCR6^+^CD4 cells in the blood. Results showed that CXCR6^+^CD4 cells did not exist in naïve *wt* mice, but were dramatically induced in the blood with EAE induction (**Figures 1A, B**). Although CXCR6^+^CD4 cells dramatically accumulated in the peripheral blood of EAE mice, *Cxcr6-ko* and *wt* mice developed comparable EAE symptom (17), suggesting that CXCR6 might only serve as a marker and be dispensable for CD4 cell chemotaxis in EAE. Since CCR6 has been well established as a chemokine receptor preferentially expressed on conventional Th17 cells (18), especially the one generated *in vitro*, we then co-stained the CCR6 and CXCR6 on draining lymph node (dLN)-derived CD4 cells at EAE onset. Strikingly, we found CXCR6^+^CD4 cells could be divided into two subpopulations: CCR6 positive and CCR6 negative ones (**Figure 1C**). We then sorted out the CCR6^+^CXCR6^-^ (as control), CCR6^+^CXCR6^+^, and CCR6^-^CXCR6^+^ effector CD4 cells from dLN at EAE onset. Q-PCR experiment showed that CCR6^+^CXCR6^+^ effector CD4 cells majorly expressed conventional Th17 signatures including *Il23r*, *Il17a* and *Rorc*, while CCR6^−^CXCR6^+^ ones majorly expressed *Ifng* and *Csf2* (encoding GM-CSF), a typical ex-Th17 phenotype (**Figure 1D**). Flow cytometry analysis further confirmed that IL-23R and IL-1R were exclusively expressed on CCR6^+^CXCR6^+^CD4 cells (**Figure 1E**).

A long-standing difficulty in treating autoimmune disease, such as MS, is the lack of understanding of inflammatory tissue-infiltrating CD4 cells. Given that most of CNS-infiltrating CD4 cells are CXCR6 positive, we compared the transcriptome profile between CNS-infiltrating CD4 cells and peripheral effector CD4 cells in EAE. We sorted out the spinal cord-infiltrated CD4 cells, and compared them with effector CD4 from dLN at EAE onset and discovered that all of *Il17a*, *Ccr6*, and *Il23r* were downregulated in spinal cord-infiltrated CD4 cells, suggesting a terminal differentiated effector status. Interestingly, we found that CD4 cells in the spinal cord were cytotoxic by highly expressing Granzyme(s) and cognate serine protease inhibitor Serpin(s) along with IFNγ and GM-CSF (**Figure 1F**). Indeed, this finding matches with the human MS study that CD4 cells from human secondary progressive MS autopsy brain samples are cytotoxic by co-expression of Eomes and Granzyme-B (19).

**Figure 2:**
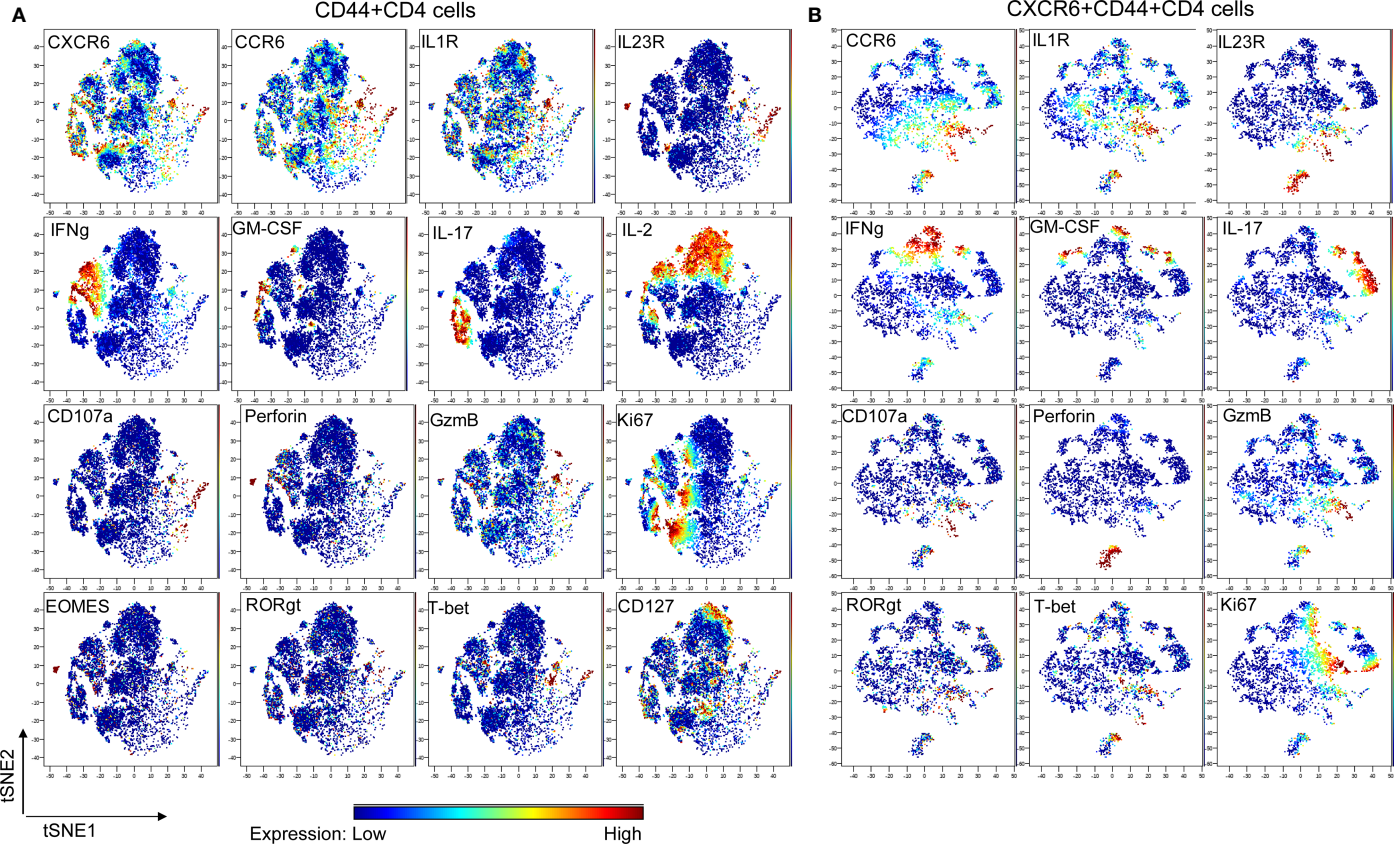
CyTOF study. *Wt* mice were induced to develop EAE and sacrificed at the disease onset (N = 9; dLN from every 3 mice were pooled and deemed as one biological sample). Lymphocytes were stimulated with PMA & Ionomycin for 4 h in the presence of Brefeldin A, then were stained with metal-conjugated mAbs and subjected to CyTOF analysis. CXCR6 mAb and CD107a mAb were added during the stimulation. Shown are viSNE plots of CyTOF of effector CD4 **(A)** and CXCR6^+^ effector CD4 **(B)** of one of 3 biological samples with the same pattern. The color indicates expression level of labeled marker.

To further confirm the existence of distinct subpopulation within CXCR6^+^CD4 cells in the periphery at EAE onset, cytometry by time of flight (CyTOF) was applied (20), which is a powerful discovery tool in identifying rare cell population and cluster. A CyTOF study of 22 markers for effector CD4 cells showed that all of IFNγ (IL-2 negative portion), GM-CSF, IL-17, IL-23R, CD107a, Granzyme-B, IL-1R1 and CCR6 and partial of T-bet and RORγt overlapped with CXCR6 expression, suggesting CXCR6 as a marker for pathogenic CD4 cells (**Figure 2A**). We then focused on CXCR6^+^CD44^+^CD4 cells, and finally confirmed that they contained a distinct CCR6 positive subpopulation, which was enriched with conventional Th17 molecules (IL-1R, IL-17, IL-23R, and RORγt), and also highly cytotoxic by expressing Perforin, CD107a and Granzyme-B (**Figure 2B**). We observed partial overlap among IFNγ, IL-17 and GM-CSF, which might suggest the co-existence of conventional Th17 and ex-Th17 cells. Interestingly, IFNγ staining did not match with T-bet well, deemed as Th1 transcriptional factor responsible for IFNγ expression. Similarly, IL-17 staining did not match with RORγt well. This discrepancy between cytokines and corresponding transcriptional factors might be due to that PMA and ionomycin stimulation will preferentially accumulate the cytokine, not the transcriptional factors, for detection. It also could be due to the complex roles of transcriptional factors involved in the CD4 cell plasticity in autoimmunity. Purified PBMC from the peripheral blood were subjected to CyTOF analysis in parallel and showed identical profile as dLN (data not shown).

## Discussion

Recently, we identified that chemokine receptor CXCR6 was preferentially expressed on pathogenic CD4 cells in EAE model (16), which i) secreted inflammatory cytokine IFNγ and GM-CSF, ii) contained cytotoxic granules, and iii) proliferated rapidly. In the current study, we further revealed that CXCR6^+^CD4 cells in EAE mice were heterogeneous, divided into two distinct subpopulations. One was CCR6 positive, enriched for cytotoxic signatures (Perforin, CD107a and GzmB) and conventional Th17 molecules (IL-17, IL-23R and RORγt); while the other was CCR6 negative, enriched solely for cytokine IFNγ and GM-CSF. Of note, although CCR6 has been established as a chemokine receptor of conventional Th17 cells, its role in EAE is still controversial since EAE disease varies from total absence (21, 22) to be delayed but more severe or totally normal (23, 24) in *ccr6*-deficient mice.

Regarding the EAE pathology, the current perception is that CD4 cells mainly interact with myeloid cells to indirectly induce the myelin sheath damage in EAE. It is unknown whether CD4 cells directly mediate the CNS damage and which molecule(s) mediate the process when that is the case. Although CD4 cell-derived GM-CSF is thought to be essential for EAE development, GM-CSF-deficient mice develop comparable EAE disease as wild type (*wt*) mice when regulatory T cells are depleted (25), suggesting that GM-CSF might function through peripheral priming step to mediate EAE. Indeed, the clinical trial of anti-GM-CSF antibody hasn’t been successful to date for MS. Thus, it is possible that EAE pathology is induced by GM-CSF-producing CD4 cells, rather than GM-CSF itself, and there might be other factors that are responsible for CD4 cell pathogenicity in EAE and human MS.

In this study, we discovered that CNS-infiltrated CD4 cells were highly cytotoxic by highly expressing various cytotoxic granzymes and cognate inhibitor Serpin(s). Granzyme(s) possesses broad pathological functions, such as triggering apoptosis of target cells (26), degrading the extracellular matrix (27), activating the myeloid cells (28, 29), and inducing self-inflicted cell death (30, 31). The finding that CNS-infiltrated CD4 cells highly expressed all of Gzm-A, -B, -K and endogenous inhibitor Serpin(s) strongly suggests that the pathogenic CD4 cells may use the Granzyme(s) to directly mediate CNS damage or to induce activation-induced cell death to maintain the homeostasis.

Overall, in the current study, we identified two distinct subpopulations in CXCR6^+^CD4 cells in EAE: CCR6^+^ cells (enriched for conventional Th17 signature with unexpected cytotoxic signature) and CCR6^-^ cells (enriched for cytokine IFNγ and GM-CSF, an ex-Th17 signature). The discovery of these two subpopulations makes *in vivo* generated pathogenic CD4 cells amenable for direct investigation. Our study also highlights the existence of cytotoxic protease(s) of encephalitogenic CD4 cells, which might be the mediator to directly mediate the CNS damage in MS. The relationship between Th17 programming and acquisition of cytotoxicity is not understood; the co-existence of these two functionally distinct subpopulations is elusive and intriguing, which requires the further investigations.

## Data Availability Statement

The datasets presented in this study can be found in online repositories. The names of the repository/repositories and accession number(s) can be found below: NCBI GEO, GSE192878 https://www.ncbi.nlm.nih.gov/geo/query/acc.cgi?acc=GSE192878.

## Ethics Statement

The animal study was reviewed and approved by the Institutional Animal Care and Use Committee of Boston Children’s Hospital.

## Author Contributions

Both authors, LH and KY, designed the research, did the experiment, analyzed the data and wrote the manuscript. All authors listed have made a substantial, direct, and intellectual contribution to the work and approved it for publication.

## Conflict of Interest

Author LH is a cofounder and shareholder of Edelweiss Immune Inc. Both authors declare that the research was conducted in the absence of any other commercial or financial relationships that could be construed as a potential conflict of interest.

## Publisher’s Note

All claims expressed in this article are solely those of the authors and do not necessarily represent those of their affiliated organizations, or those of the publisher, the editors and the reviewers. Any product that may be evaluated in this article, or claim that may be made by its manufacturer, is not guaranteed or endorsed by the publisher.
